# A multiomics profile of coordinated defense and key candidate genes against bacterial wilt in tobacco

**DOI:** 10.1038/s41598-026-36889-1

**Published:** 2026-01-23

**Authors:** Yu Qing, Lin Wei, Lin Yong, Wang Tao, Wu Shengxin, Cheng Yazhi, Chen Shunhui, Zhao Chengkun, Zhao Xinyi, Yu Wen

**Affiliations:** 1 Tobacco-Rice Synergistic Development Industrial Technology Engineering Center, Fujian Institute of Tobacco Agricultural Sciences, Fuzhou, 350000 China; 2 Fujian Tobacco Agricultural science institute branch, Nanping Tobacco Monopoly Bureau, Nanping, 353000 China

**Keywords:** Bacterial wilt, Transcriptome, Metabolome, Biotechnology, Genetics, Molecular biology, Plant sciences

## Abstract

Bacterial wilt, caused by Ralstonia solanacearum, is a devastating disease that limits global tobacco production. To decipher the molecular basis of resistance, we conducted an integrated multiomics analysis of a susceptible cultivar, Honghua Dajinyuan (HD), and a moderately resistant cultivar, Yanyan 97 (YY), leveraging LC‒MS-based metabolomics and RNA-seq transcriptomics. The resistant YY cultivar exhibited coordinated multitiered defense, characterized by the accumulation of resistance-related metabolites (e.g., prenol lipids and organooxygen compounds) and the enrichment of plant hormone signaling pathways. Transcriptomic analysis revealed 818 differentially expressed genes (DEGs) involved in cell wall modification and stress responses. Crucially, the integration of weighted gene coexpression network analysis (WGCNA) with prior QTL mapping pinpointed *Nta17g05760* within the qBWR17b locus as a core candidate gene. Our study systematically elucidates a defense network involving hormone signaling, cell wall reinforcement, and antimicrobial synthesis and provides a key candidate gene and theoretical foundation for the molecular breeding of wilt-resistant tobacco.

**Supplementary Information:**

The online version contains supplementary material available at 10.1038/s41598-026-36889-1.

## Indroduction

*Ralstonia solanacearum* is a highly damaging bacterial pathogen worldwide^1^. Soil-borne wilt disease severely threatens the growth of more than 450 plant species, notably those within the Solanaceae family^2,3^. The pathogen invades host plants through root wounds or natural openings, subsequently colonizing the vascular system. Extensive proliferation and secretion of extracellular polysaccharides obstruct xylem vessels, impeding water transport and ultimately leading to wilting and plant death^4,5^. Tobacco, as an important cash crop, faces significant production losses due to bacterial wilt. A comprehensive understanding of the molecular basis of resistance is, therefore, of great theoretical and practical importance.

Bacterial wilt resistance in tobacco is a complex quantitative trait whose expression is regulated by multiple genes working in concert^6,7^. The molecular basis of this trait is now accessible owing to chromosome-level reference genomes for *N. tabacum* and *N. benthamiana*^8^. In addition to genetic factors, phytohormone pathways, especially those involving salicylic acid (SA) and jasmonic acid (JA), are crucial mediators of resistance^9,10^. This complexity is reflected at the transcriptional level, where early infection triggers cultivar-specific responses enriched in signal transduction and transcriptional regulation^11^. Complementing these findings, metabolomic studies provide direct insight into the biochemical end products of defense^12^. For example, Yang et al. reported the accumulation of metabolites, including β-alanine, phenylalanine, and leucine, in infected tobacco plants^13^.

Previous research has identified key components of tobacco defense against bacterial wilt at both the transcriptional and metabolic levels. However, these efforts often lack integration, preventing a comprehensive picture of the interconnected regulatory landscape. Therefore, we employed an integrated transcriptome and metabolome analysis to compare the dynamic responses of resistant (Yanyan 97, YY97) and susceptible (Honghua Dajinyuan, HD) tobacco cultivars to *R. solanacearum* challenge. This study aims to systematically identify critical genes and metabolites within the resistance network, thereby providing a foundational framework for future functional validation and breeding applications.

## Results

### Field incidence of bacterial wilt in varieties with different levels of resistance under natural infection conditions

**Fig. 1:**
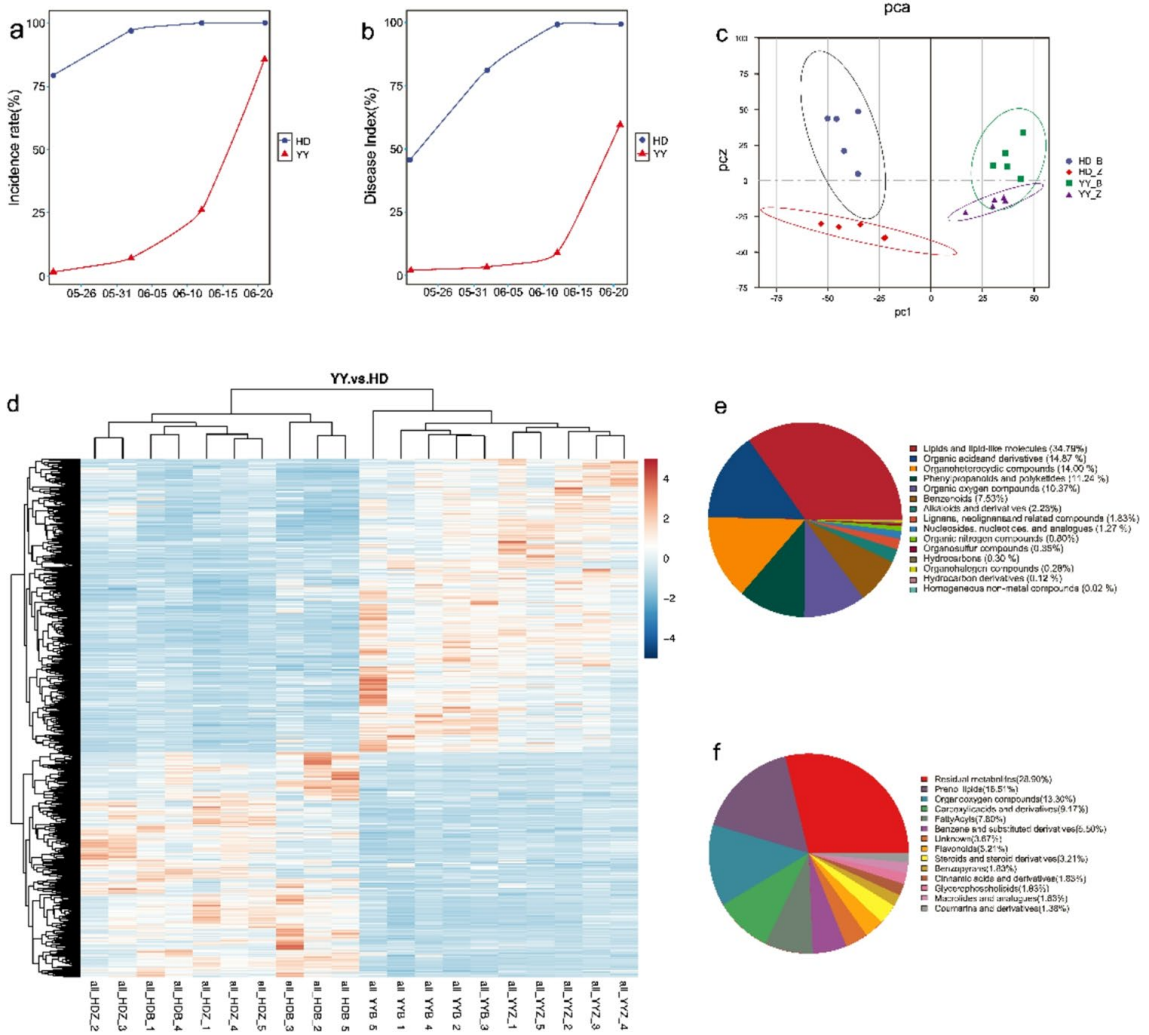
Field disease survey and untargeted metabolomics analysis. (**a**, **b**) Temporal dynamics of (**a**) incidence rate and (**b**) disease index for the HD (susceptible) and YY (resistant) cultivars in the field. (**c**) PCA score plot of metabolic profiles. (**d**) Heatmaps of DAMs compared between different group. (**e**) Classification of all detected metabolites. (**f**) Classification of differentially accumulated metabolites (DAMs).

To evaluate the field resistance of the two tobacco cultivars to bacterial wilt, Honghua Dajinyuan (HD, susceptible) and Yanyan 97 (YY, moderately resistant) were planted in a disease nursery for natural infection. The disease assessment revealed a stark contrast in resistance between the two cultivars (Fig. 1a,b). The susceptible cultivar HD reached 100% incidence, with a disease index (DI) of 100% by June 21. In contrast, YY significantly delayed disease progression, with a final incidence rate of 85.51% and a DI of 59.33% on the same date. This pronounced difference in phenotypic resistance confirms the better defensive capacity of the YY cultivar against bacterial wilt.

### Metabolomic analysis

#### Effects of bacterial wilt on tobacco metabolism

To investigate the metabolic basis of resistance, we performed nontargeted metabolomic profiling (LC‒MS) on healthy and infected leaves of the resistant cultivar Yanyan 97 (YY) and the susceptible cultivar Honghua Dajinyuan (HD). A total of 1,483 metabolites were identified and categorized into 15 classes (Fig. 1d,e). Lipids and lipid-like molecules constituted the most abundant category, comprising 534 metabolites (34.79%) (Fig. 1e). Principal component analysis (PCA) revealed that the metabolic profiles of the YY and HD samples formed distinct clusters separated primarily along the first principal component (PC1), irrespective of their infection status (Fig. 1c). This clear separation indicates fundamental differences in the inherent metabolic backgrounds of the two cultivars.

**Fig. 2:**
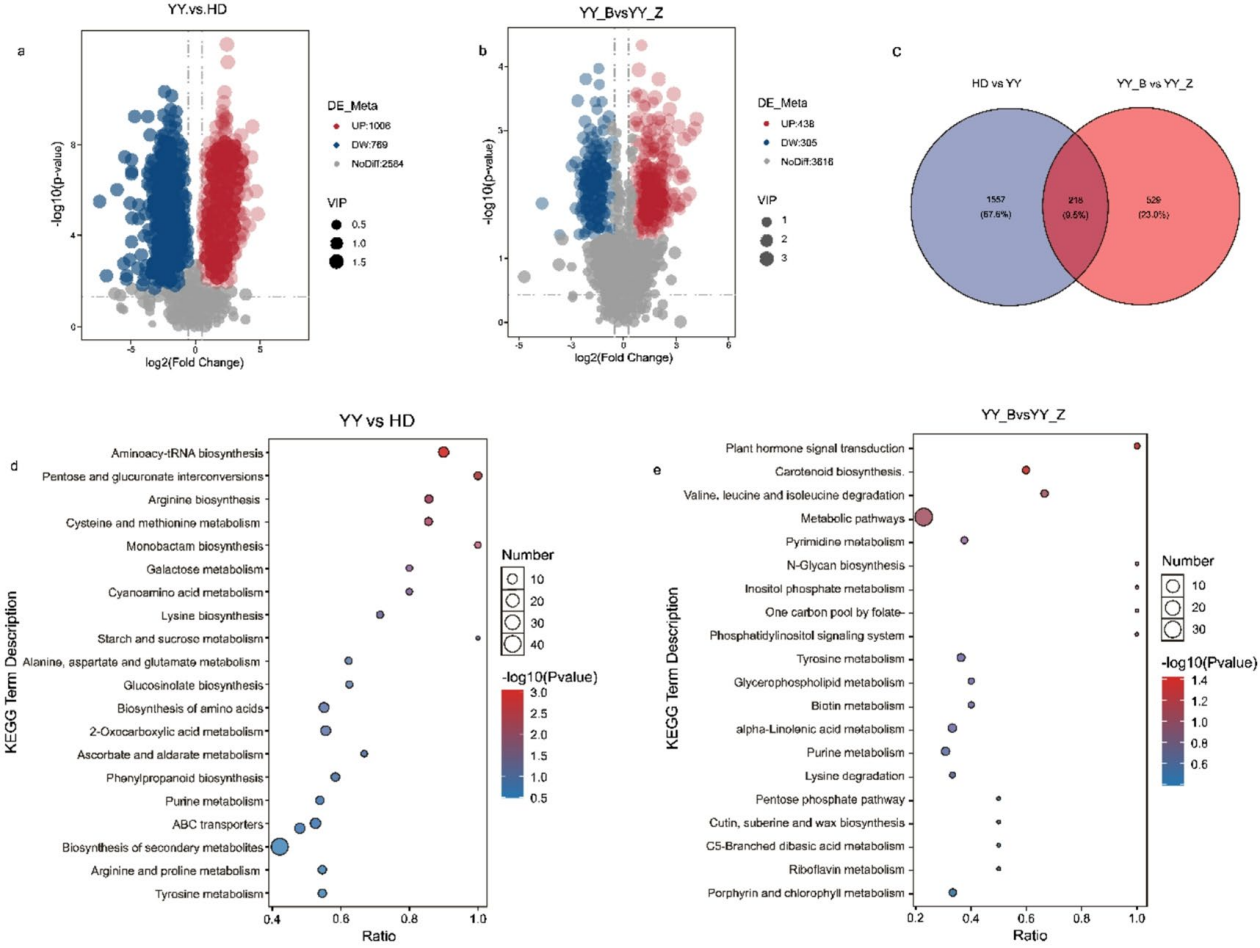
Analysis of differentially accumulated metabolites (DAMs) under bacterial wilt stress. (**a**, **b**) Volcano plots of DAMs from (**a**) cultivar comparison and (**b**) YY infection response. (**c**) Venn diagram showing the overlap of DAMs between the two key comparisons. (**d**, **e**) KEGG enrichment results for DAMs in the (**d**) YY vs. HD and (**e**) YY_B vs. YY_Z comparisons.

We identified multiple sets of differentially accumulated metabolites (DAMs) using the thresholds of FC ≥ 1.5 or FC ≤ 0.67, VIP > 1, and *p* < 0.05. Within the resistant cultivar YY, infection (YY_B vs. YY_Z) induced significant changes in 743 DAMs (438 upregulated, 305 downregulated). A broader comparison between the cultivars (YY vs. HD) revealed 1,775 DAMs (1,006 upregulated, 769 downregulated) (Fig. 2a, b). To pinpoint metabolites specifically associated with the resistance response in YY, we performed a Venn analysis of these two DAM sets, which identified 218 YY-specific response metabolites (Fig. 2c). The classification of these key metabolites revealed that they were primarily prenol lipids, organooxygen compounds, carboxylic acids and derivatives, fatty acyls, and benzene and substituted derivatives (Fig. 1f).

#### KEGG pathway enrichment analysis

To decipher the metabolic basis of resistance, we conducted KEGG enrichment analysis on the differentially accumulated metabolites (DAMs). The results revealed that DAMs in the resistant cultivar YY following infection (YY_B vs. YY_Z) were significantly enriched in ‘plant hormone signal transduction’, further analysis revealed that the DAMs were also enriched in two specific biosynthesis pathways: ‘alpha-Linolenic acid metabolism’ (jasmonate precursors) and ‘Carotenoid biosynthesis’ (ABA precursors), suggesting the potential involvement of JA and ABA signaling. (Fig. 2d,e). Moreover, DAMs that constitutively differentiated YY from HD (YY vs. HD) were enriched in fundamental metabolic pathways such as ‘Aminoacyl-tRNA biosynthesis’, ‘Arginine biosynthesis’, ‘Cysteine and methionine metabolism’, and ‘Pentose and glucuronate interconversions’. Intersection of the enriched pathways from both comparisons highlighted ‘Tyrosine metabolism’ and ‘Purine metabolism’ as common components of the response.

### Transcriptomic analysis

#### Transcriptome sequencing and DEG identification

**Fig. 3:**
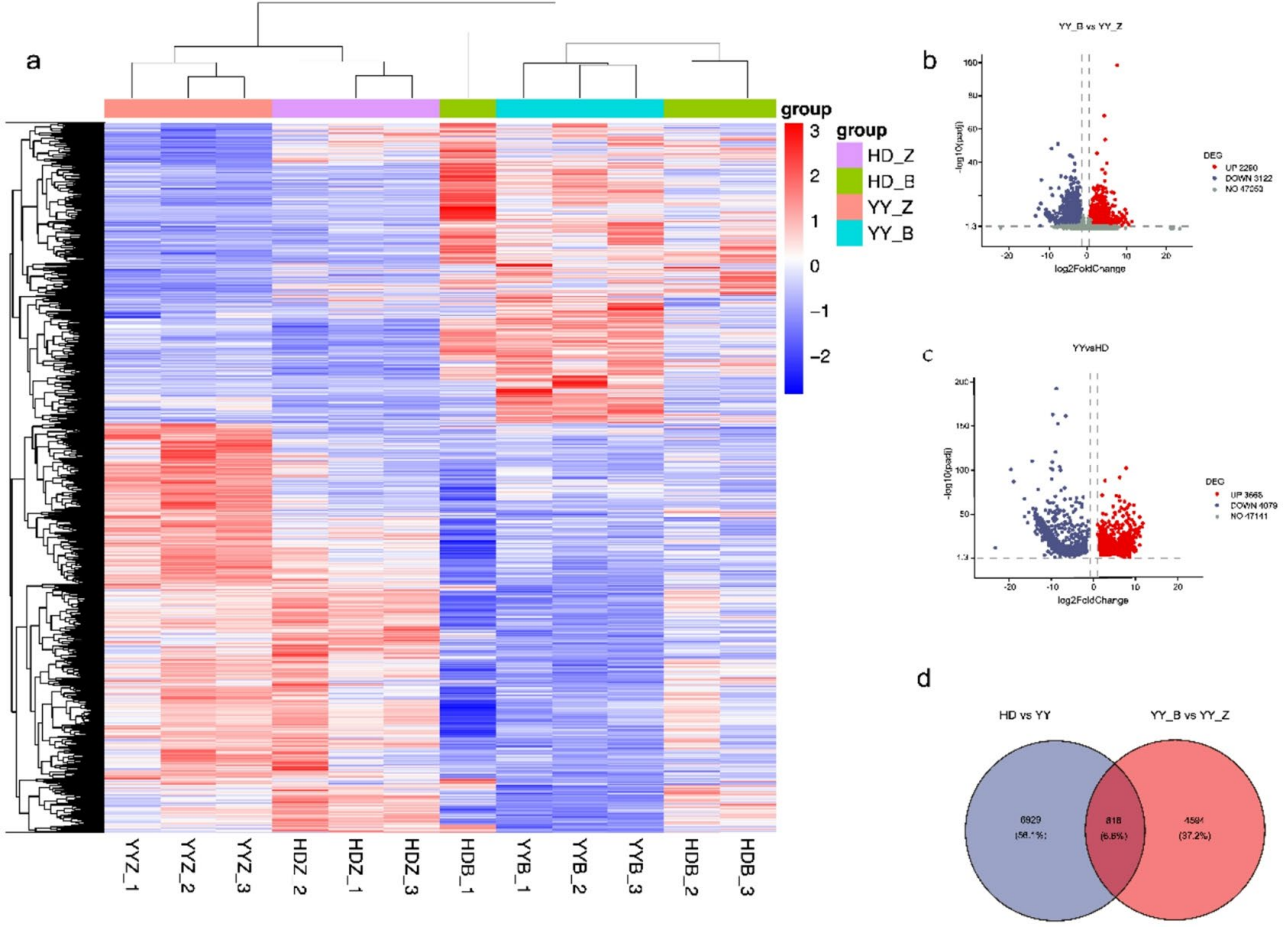
Transcriptome analysis of tobacco varieties with contrasting resistance to bacterial wilt. (**a**) Shown is a heatmap of DEGs, with hierarchical clustering of samples and genes. (**b**, **c**) Presented are volcano plots of DEGs for the (**b**) YY_B vs. YY_Z and (**c**) YY vs. HD comparisons. (**d**) Displayed is a Venn diagram identifying common and unique DEGs across the two key comparisons.

Transcriptome sequencing of the leaf samples generated approximately 87.18 Gb of high-quality clean data. The sequencing quality was robust, with minimum Q20 and Q30 scores of 99.28% and 96.78%, respectively, confirming the reliability of the data for downstream analysis (Supplementary Table 1). A global view of gene expression patterns, illustrated by a heatmap, revealed clear separation between samples on the basis of both infection status and cultivar (Fig. 3a). Differential expression analysis revealed 5,412 genes (|log₂FC| ≥ 1, *p* < 0.05) responsive to infection in the resistant cultivar YY (YY_B vs. YY_Z), with 2,290 upregulated genes and 3,122 downregulated genes. A comparison between the cultivars (YY vs. HD) revealed 7,747 differentially expressed genes (3,668 upregulated, 4,079 downregulated) (Fig. 3b,c). A Venn diagram analysis further revealed 818 DEGs that were common to both comparisons, representing a core set associated with YY resistance (Fig. 3d). To validate the RNA-seq findings, we selected three key differentially expressed genes (DEGs) (*Nta05g03660*, *Nta17g04700*, and *Nta17g05760*) for quantitative PCR (qPCR) analysis. As shown in Supplementary Fig. 1, the expression levels of these candidate genes were significantly lower in the resistant cultivar YY than in the susceptible cultivar HD under the same conditions. This result is consistent with our transcriptomic data, thereby confirming the reliability of the RNA-seq results.

#### GO and KEGG pathway analysis

**Fig. 4:**
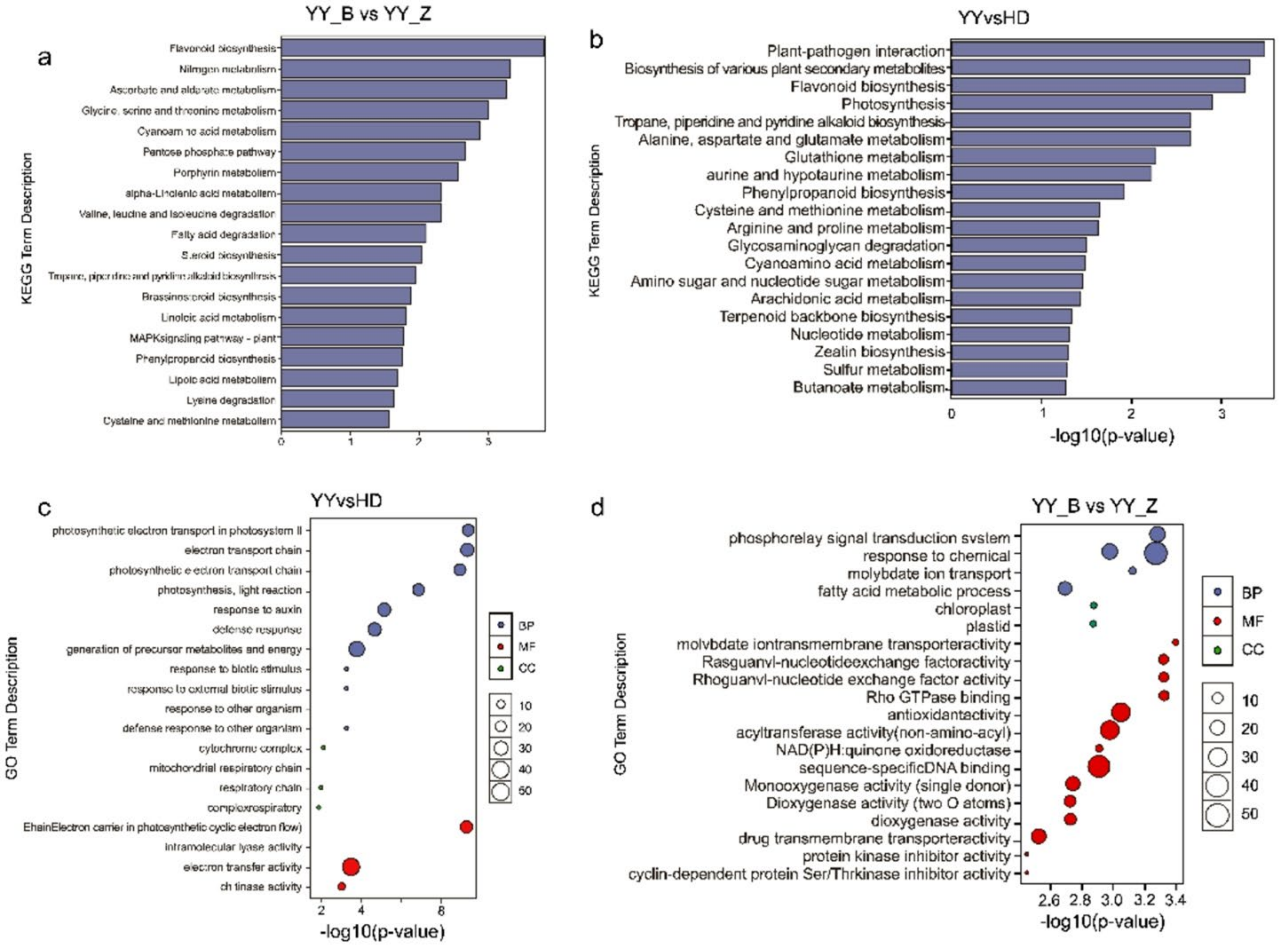
Functional enrichment analysis of differentially expressed genes (DEGs). (**a**, **b**) KEGG pathway enrichment analysis for the (**a**) YY_B vs. YY_Z and (**b**) YY vs. HD comparisons. The y-axis represents the pathway name, and the x-axis represents the enrichment significance (-log10(p value)). (**c**, **d**) Gene Ontology (GO) enrichment analysis for the (**c**) YY vs. HD and (**d**) YY_B vs. YY_Z comparisons. The results are summarized for the three main GO categories: Biological Process (BP), Cellular Component (CC), and Molecular Function (MF).

We performed Kyoto Encyclopedia of Genes and Genomes (KEGG) pathway enrichment analysis on the DEGs to gain functional insights into the resistance mechanism. The results revealed significant differences between the various comparisons (Fig. 4a,b). Genes that were constitutively differentially expressed between the resistant and susceptible cultivars (YY vs. HD) were enriched in broad defense and metabolic pathways, including ‘plant‒pathogen interaction’. Moreover, genes specifically induced by infection in the resistant YY cultivar (YY_B vs. YY_Z) were enriched in more specific metabolic processes, such as ‘flavonoid biosynthesis’, ‘ascorbate and aldarate metabolism’, and ‘nitrogen metabolism’.

Gene Ontology (GO) enrichment analysis provided functional insights into the differentially expressed genes (DEGs) (Fig. 4c,d). The DEGs identified from the cultivar comparison (YY vs. HD) were enriched predominantly in GO terms related to energy metabolism (e.g., ‘photosynthetic electron transport chain’, ‘electron transport coupled to ATP synthesis’) and the response to stress. In contrast, the DEGs related to the infection response in YY (YY_B vs. YY_Z) were significantly enriched in terms associated with signal transduction (e.g., ‘cation transmembrane transporter activity’ and ‘response to chemicals’) and the antioxidant system (e.g., ‘antioxidant activity’ and ‘oxidoreductase activity’). The GO terms common to both comparisons were involved primarily in cell wall modification (e.g., ‘aminoglycan catabolic process’, ‘cell wall macromolecule catabolic process’) and response to various stimuli.

#### Analysis of the weighted gene coexpression network

**Fig. 5:**
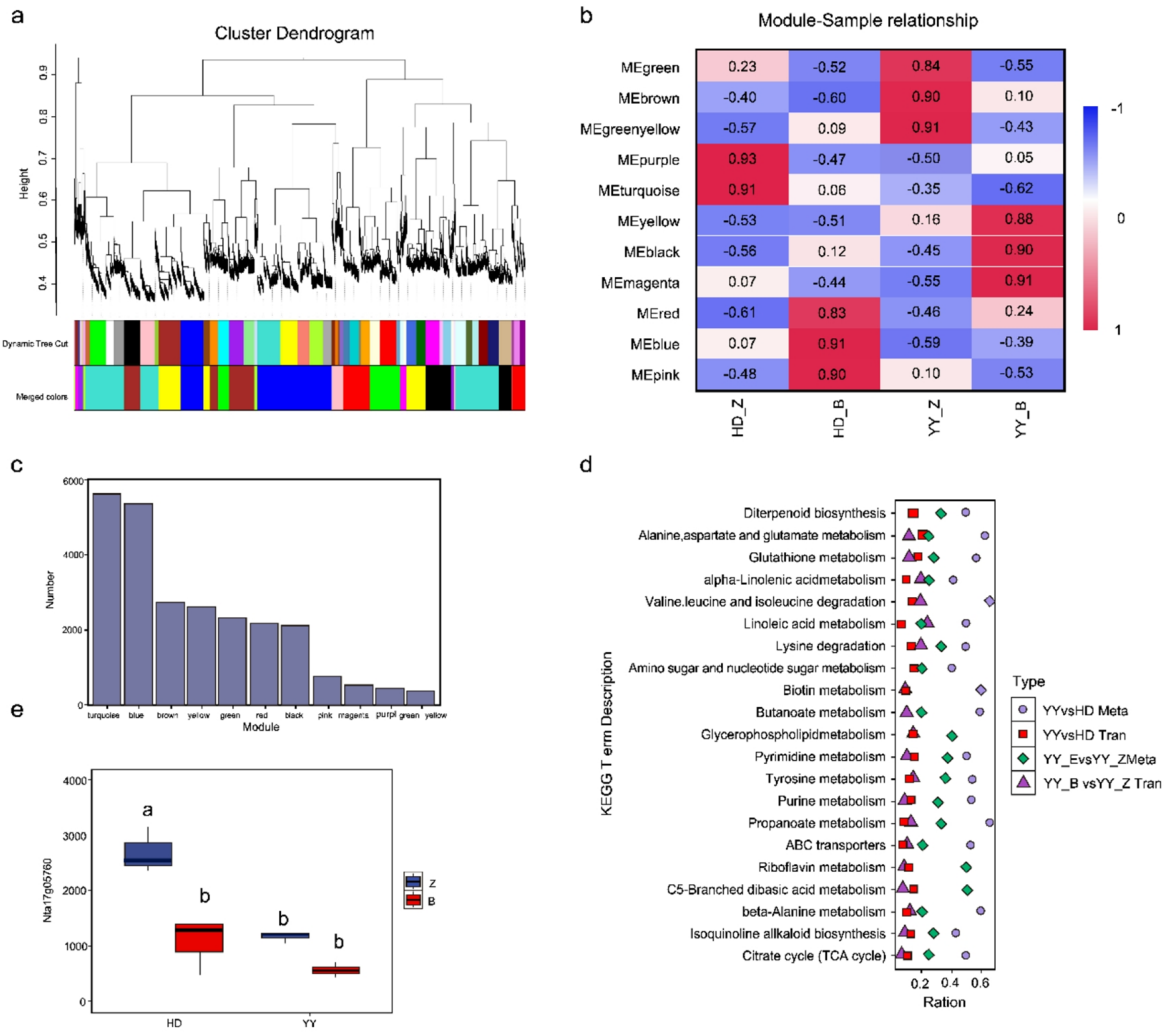
Weighted gene coexpression network analysis (WGCNA) and multiomics integration in tobacco under bacterial wilt stress. (**a**) Gene clustering dendrogram and identified coexpression modules. (**b**) Correlation heatmap between module eigengenes and sample groups. Color intensity indicates the Pearson correlation coefficient. (**c**) Bar chart of gene numbers in each coexpression module. (**d**) KEGG pathways commonly enriched for both differentially expressed genes (DEGs) and differentially accumulated metabolites (DAMs). (**e**) Expression of the core candidate gene *Nta17g05760* across sample groups (Different letters indicate significant differences).

To decipher the coordinated transcriptional regulatory mechanisms underlying the response to *Ralstonia solanacearum*, we performed weighted gene coexpression network analysis (WGCNA). This analysis clustered genes with highly correlated expression patterns across all samples into coexpression modules. A total of 11 distinct modules were identified, encompassing approximately 25,000 genes (Fig. 5a). The two largest modules were the turquoise module, containing 5,647 genes, and the blue module, containing 5,373 genes, which were identified as core functional units for subsequent analysis (Fig. 5c).

The correlation between module eigengenes and sample traits revealed a significant association pattern (Fig. 5b). Specifically, the expression profiles of both the blue and turquoise modules were positively correlated with the susceptible cultivar Hongda (HD) and negatively correlated with the resistant cultivar Yanyan 97 (YY). Furthermore, an assessment of intramodular connectivity indicated that they may function as cohesive units in the regulatory network (Supplementary Table 2).

By intersecting the gene lists from the differential expression analysis with the two key WGCNA modules (blue and turquoise), we identified 57 candidate genes in the blue module and 81 in the turquoise module that were also differentially expressed. Among these genes, the expression pattern of *Nta17g05760* was particularly notable. Under healthy conditions (Z), *Nta17g05760* expression was 2.3-fold higher in HD than in YY (Fig. 5e). After infection, HD expression decreased significantly by 59.7%, reaching a level comparable to YY, whose expression change was not significant. This dynamic expression pattern is consistent with a role for *Nta17g05760* in the pathogen-induced immune response.

### Transcriptome‒metabolome correlation analysis

To integrate the transcriptomic and metabolomic data, we performed correlation analyses for two key comparisons: YY_B vs. YY_Z and YY vs. HD. These analyses revealed 13,159 differentially expressed genes (DEGs) and 2,522 differentially accumulated metabolites (DAMs) (Figs. 2c and 3d). Significant correlations were observed between the majority of DEGs and DAMs in both comparisons (Supplementary Tables 3, 4), suggesting widespread regulatory interactions between transcriptional and metabolic responses. Furthermore, we identified KEGG pathways that were concurrently enriched in both the DEG and DAM datasets. This crossomics integration revealed 21 shared pathways (Fig. 5d), which were involved primarily in metabolic processes, including ‘Diterpenoid biosynthesis’ and ‘Alanine, aspartate and glutamate metabolism’. The coenrichment of these pathways highlights their potential central role in coordinating the defense response to *R. solanacearum*.

## Discussion

Integrated multiomics profiling of resistant and susceptible tobacco cultivars challenged with *Ralstonia solanacearum* demonstrated that effective resistance involves a coordinated multitiered defense system. This system involves synergistic activation of signal transduction pathways, antioxidant systems, and energy metabolism, and our analysis identified core candidate genes orchestrating this response.

To decipher the molecular basis of the differential defense response, our nontargeted metabolomics identified 743 DAMs in the resistant YY cultivar post-infection, and a key finding was the specific induction of the diterpenoid biosynthesis pathway in the resistant YY cultivar. While diterpenoids are well known contributors to tobacco aroma^14^, their significant enrichment upon *Ralstonia solanacearum* infection suggests a previously underappreciated role in pathogen defense. This finding is consistent with the established literature showing that diterpenoid compounds possess broad antimicrobial and signaling activities that contribute to plant immunity^15,16^. Our metabolic data highlight the importance of hormone signaling in resistance. The specific enrichment of JA- and ABA-related biosynthetic pathways in YY upon infection suggests the activation of these hormonal pathways. This aligns with the established role of JA in broad-spectrum defense^10^ and extends it to Ralstonia resistance. Coupled with the supportive evidence for ABA^17^, our findings implicate a coordinated crosstalk between JA and ABA as a potential mechanism for effective resistance.

The lab previously constructed a linkage map comprising 201 simple sequence repeat (SSR) markers from an F_2_ population derived from HD and YY, with a total length of 2326.7 cM^18,19^. This successfully mapped multiple QTL loci associated with bacterial wilt, but the underlying causal genes and their molecular functions remained uncharacterized. To identify key regulators, we focused on 138 hub genes from the resistance-associated blue and turquoise WGCNA modules. This study identified the gene Nta17g05760 located at the qBWR17b locus as a high-confidence candidate gene. Notably, the relative expression level of Nta17g05760 was significantly lower in the resistant YY cultivar compared to HD. Furthermore, its expression remained significantly downregulated following infection in YY, suggesting it may function as an inhibitor of the defence response.

## Conclusions

By integrating transcriptomic and metabolomic analyses, this study systematically deciphered the multi-layered defense mechanisms against bacterial wilt in tobacco, encompassing both metabolic and transcriptional regulation. Crucially, we precisely localized core candidate genes (*Nta17g05760*) within a known quantitative trait locus (qBWR17b), thereby successfully bridging QTL mapping with concrete gene discovery. These findings provide crucial insights and precise molecular targets for advancing the molecular breeding of resistant tobacco varieties. However, as the present work is primarily confined to correlative molecular profiling, future research should focus on functional validation, such as constructing mutant or overexpression lines of the candidate genes, to definitively establish their roles in disease resistance.

## Methods

### Field trial design and sample collection

This study employed the susceptible tobacco cultivar ‘Honghua Dajinyuan’ (HD) and the moderately resistant cultivar ‘Yanyan 97’ (YY). The plants were arranged in a randomized block design in a field with a natural incidence of bacterial wilt and were transplanted on March 12, 2025. Conventional field management was applied. At the disease peak (June 9, 2025), leaf samples were collected from both symptomatic (B) and asymptomatic (Z) plants of each cultivar, and four sample groups were defined: YY_B, YY_Z, HD_B, and HD_Z. Each group included biological replicates (*n* ≥ 3). All the samples were flash-frozen in liquid nitrogen and stored at −80 °C for subsequent multiomics analyses.

### Survey of bacterial wilt disease severity and resistance evaluation

Disease progression was monitored through field surveys following the natural onset of bacterial wilt. The incidence rates and disease indices were assessed on four dates (May 22, June 2, June 12, and June 21, 2025) to capture the dynamics of infection. For each cultivar, a minimum of ten replicate plots were evaluated per observation.

The disease index (DI) is calculated via the following formula^20^:

$$DI = \sum {\frac{{(Number\:of\:plants\:with\:disease\:rating\: \times \:\:Disease\:rating\,value)}}{{(Total\:number\:surveyed \times \:Highest\:disease\:rating)}}} \times 100$$

The resistance level of varieties was evaluated on the basis of the final disease index.

### Metabolomics analysis

Metabolite extraction and LC‒MS analysis were performed as follows. Leaf tissues from infected (B) and healthy control (Z) plants of both the HD and YY cultivars (*n* = 5 biological replicates per group) were collected at the peak of the disease (June 9). The frozen samples were finely ground to powder in liquid nitrogen. Approximately 50 mg of powdered tissue was homogenized with 500 µL of prechilled 80% methanol aqueous solution. The mixture was vortexed thoroughly, incubated on ice for 5 min, and then centrifuged at 15,000 × g for 20 min at 4 °C. A precise volume of the resulting supernatant was diluted with mass spectrometry-grade water to a final methanol concentration of 53%. This diluted extract was centrifuged again under the same conditions (15,000 × g, 20 min, 4 °C). The final supernatant was transferred to an LC‒MS vial for subsequent analysis. Liquid chromatography‒mass spectrometry (LC‒MS)^21^ was conducted by Novogene Co., Ltd. (Beijing, China).

Metabolite annotation was performed by cross-referencing the identified compounds against the KEGG^22^, HMDB, and LIPIDMAPS databases. For multivariate statistical analysis, the raw data were processed via the metaX software toolkit to perform principal component analysis (PCA) and partial least squares-discriminant analysis (PLS-DA). The variable importance in projection (VIP) scores from the PLS-DA model were extracted for each metabolite. For univariate analysis, Student’s t test was applied to compare metabolite levels between groups, yielding p values. The fold change (FC) in abundance was also calculated. Metabolites were defined as significantly differentially accumulated on the basis of the following combined criteria: a VIP score > 1, a p value < 0.05, and an |log2(fold change)| > 1.

### Transcriptome analysis

Transcriptome sequencing was conducted to profile gene expression changes in response to bacterial wilt infection. Total RNA was extracted from leaf samples of infected (B) and healthy control (Z) plants from both the HD and YY cultivars (*n* = 3 biological replicates per group) at the peak disease stage (June 9). All RNA extraction and library preparation were performed by Novogene Co., Ltd. (Beijing, China). Sequencing was carried out on an Illumina NovaSeq 6000 platform^23^. The resulting raw reads were aligned to the reference genome, and gene-level read counts were quantified via featureCounts (v1.5.0-p3). Gene expression levels were estimated as fragments per kilobase of transcript per million mapped reads (FPKM) values. Differential expression analysis between groups was performed via the DESeq2 package (v1.20.0) with a threshold of |log2(fold change)| > 1 and an adjusted p value < 0.05. Gene Ontology (GO) and Kyoto Encyclopedia of Genes and Genomes (KEGG) pathway enrichment^22^ analyses of the differentially expressed genes (DEGs) were conducted via the clusterProfiler package (v3.8.1).

Following reverse transcription of the RNA samples, quantitative PCR (qPCR) was performed using a standard two-step protocol on a [QuantReady K9600] real-time PCR system. The thermal cycling conditions were as follows: initial denaturation at 95 °C for 30 s; followed by 40 cycles of denaturation at 95 °C for 5 s, annealing at 61 °C for 36 s, and extension at 72 °C for 1 min. *NbEF1a*^24^ was used as an internal reference gene for normalization. Relative gene expression levels were calculated using the comparative 2^(–ΔΔCt) method. All primer sequences are provided in Supplementary Table 5.

### Metabolomic and transcriptomic coexpression jint analysis

This study employed Pearson’s correlation coefficient from the mixOmics package in R for association analysis, calculating the correlation coefficient r² and p values for differentially expressed genes and metabolites. All differentially expressed genes and metabolites identified in the YY_B vs. YY_Z and YY vs. HD comparisons were mapped to the KEGG database to obtain their shared pathway information and conduct statistical analysis.

### Statistical analysis

Data analysis and visualization were conducted in R and Microsoft Excel 2010. All experiments included a minimum of three biological replicates (*n* ≥ 3). Differences between means were assessed by one-way ANOVA with the least significant difference (LSD) post hoc test. Statistical significance was defined as *p* < 0.05.

## Supplementary Information

Below is the link to the electronic supplementary material.

Supplementary Material 1

Supplementary Material 2

## Data Availability

The datasets generated and analysed during the current study are available in the NCBI repository under accession number PRJNA1347637 [https://dataview.ncbi.nlm.nih.gov/object/PRJNA1347637] and the MetaboLights repository under accession number MTBLS13209 [https://www.ebi.ac.uk/metabolights/reviewer342a2bce-c769-45b8-ae18-de86463ae962].
