# Supplementary material for: A multiomics profile of coordinated defense and key candidate genes against bacterial wilt in tobacco
Source: Sci Rep. 2026 Jan 23;16:6043. doi: 10.1038/s41598-026-36889-1 (PMC12902097; doi:10.1038/s41598-026-36889-1)
Supplement: Supplementary file 1 — Supplementary Material 1 [file 41598_2026_36889_MOESM1_ESM.docx]

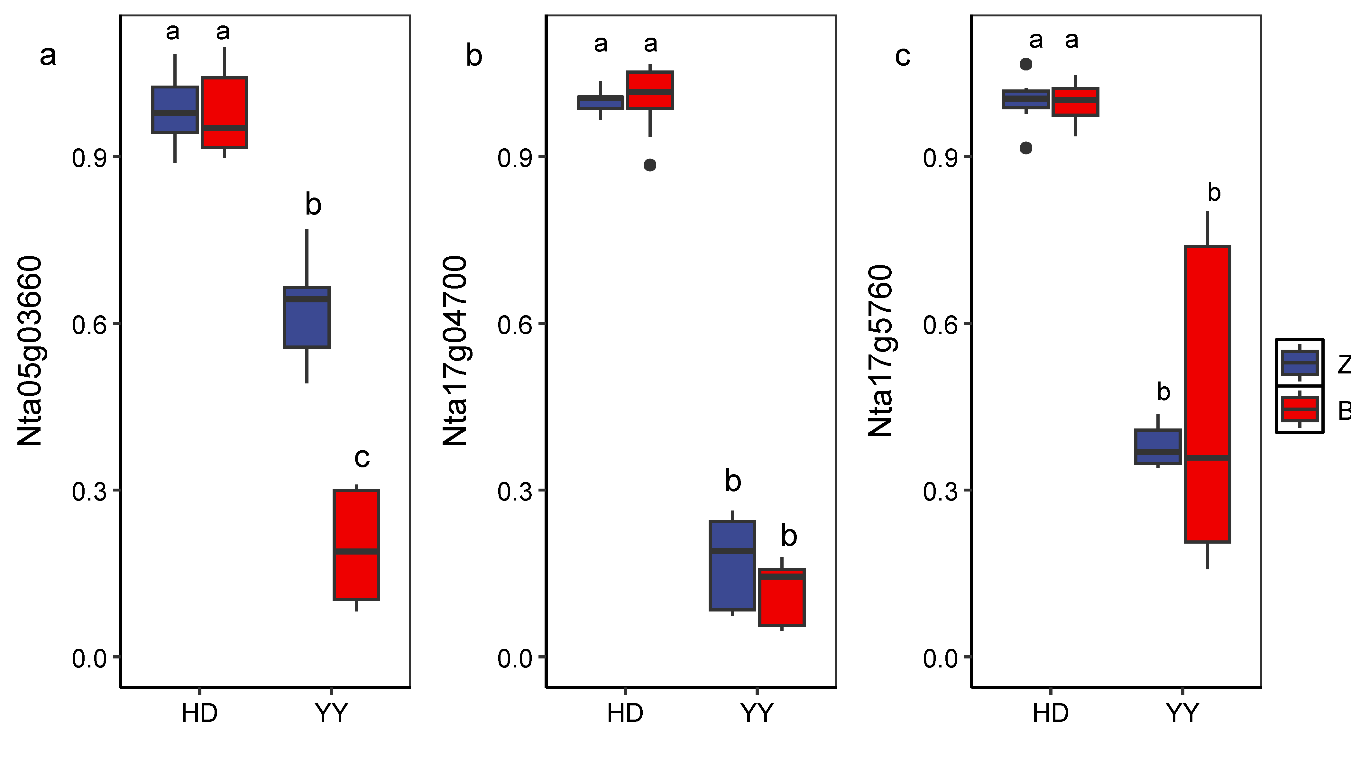
 Supplementary Fig. 1. Expression validation of candidate differentially expressed genes (DEGs).

(a-c) Expression levels of three candidate DEGs assessed by qRT-PCR: (a) Nta05g03660, (b) Nta17g04700, (c) Nta17g05760. Statistical significance between groups is indicated by different lowercase letters (p < 0.05, two-way ANOVA with LSD post-hoc test).
